# A Built-In CpG Adjuvant in RSV F Protein DNA Vaccine Drives a Th1 Polarized and Enhanced Protective Immune Response

**DOI:** 10.3390/v10010038

**Published:** 2018-01-15

**Authors:** Yao Ma, Yue-Ying Jiao, Yun-Zhou Yu, Nan Jiang, Ying Hua, Xiu-Juan Zhang, Yuan-Hui Fu, Xiang-Lei Peng, Yan-Peng Zheng, Larry J. Anderson, Jin-Sheng He

**Affiliations:** 1 College of Life Sciences & Bioengineering, Beijing Jiaotong University, Beijing 100044, China; 13118422@bjtu.edu.cn (Y.M.); 11118411@bjtu.edu.cn (Y.-Y.J.); 14121579@bjtu.edu.cn (N.J.); 09118370@bjtu.edu.cn (Y.H.); 12118414@bjtu.edu.cn (X.-J.Z.); yhfu@bjtu.edu.cn (Y.-H.F.); xlpeng@bjtu.edu.cn (X.-L.P.); ypzheng@bjtu.edu.cn (Y.-P.Z.); 2 Department of Pediatrics, Emory University and Children’s Healthcare of Atlanta, Atlanta, GA 30322, USA; 3 Beijing Institute of Biotechnology, Beijing 100071, China; yunzhouyu@163.com

**Keywords:** human respiratory syncytial virus, CpG oligodeoxynucleotide, DNA vaccine, built-in adjuvant, protective immunity

## Abstract

Human respiratory syncytial virus (RSV) is the most significant cause of acute lower respiratory infection in children. However, there is no licensed vaccine available. Here, we investigated the effect of five or 20 copies of C-Class of CpG ODN (CpG-C) motif incorporated into a plasmid DNA vaccine encoding RSV fusion (F) glycoprotein on the vaccine-induced immune response. The addition of CpG-C motif enhanced serum binding and virus-neutralizing antibody responses in BALB/c mice immunized with the DNA vaccines. Moreover, mice vaccinated with CpG-modified vaccines, especially with the higher 20 copies, resulted in an enhanced shift toward a Th1-biased antibody and T-cell response, a decrease in pulmonary pathology and virus replication, and a decrease in weight loss after RSV challenge. This study suggests that CpG-C motif, cloned into the backbone of DNA vaccine encoding RSV F glycoprotein, functions as a built-in adjuvant capable of improving the efficacy of DNA vaccine against RSV infection.

## 1. Introduction

Human respiratory syncytial virus (RSV) is an enveloped, non-segmented, negative-sense, single-stranded RNA virus in the *Pneumoviridae* family. It causes respiratory disease throughout life with greatest disease burden in infants and the elderly [1,2]. RSV is estimated to cause 30 million lower respiratory tract infections and at least 60,000 deaths worldwide each year in children <5 years of age [3]. Despite RSV being discovered over 60 years ago, an effective vaccine is still unavailable [4,5,6]. The first vaccine, a formalin-inactivated, alum adjuvanted RSV (FI-RSV), was evaluated in infants and young children in the 1960s. Unfortunately, this vaccine caused enhanced respiratory disease (ERD) resulting in a high rate of hospitalization and two deaths associated with peribronchiolar mononuclear cell infiltration with an excess of eosinophils [7,8,9,10]. Two features of the FI-RSV vaccine that may have contributed to ERD were induction of antibodies with poor neutralizing activity and a Th2 polarized memory response [11,12,13]. The poor neutralizing activity of FI-RSV-induced antibodies may have resulted from lack of pre-fusion F epitopes on the viral surface. A Th2 polarized immune response with enhanced pulmonary inflammation including eosinophilia has been a consistent feature of RSV challenged FI-RSV vaccinated animals [14,15,16]. This experience with the FI-RSV vaccine and subsequent studies suggest that a vaccine should induce antibodies with good neutralizing activity and a Th1 rather than a Th2-biased memory response.

Of the three RSV envelope glycoproteins, the attachment glycoprotein (G), the fusion glycoprotein (F), and a small hydrophobic protein (SH), the F protein is most effective at inducing protective immunity. The F protein supports viral attachment, is responsible for fusion and entry, and elicits a high level of neutralizing antibodies. Furthermore, it is more conserved and induces better cross-protection against different RSV strains than the G protein and the other surface protein [17,18,19].

Since DNA vaccines produce antigens intracellularly, they induce antigen-specific humoral and cellular immune responses in a fashion similar to a live virus infection. Since live virus infection does not lead to ERD, it is felt that given the similarity in the way immunity is induced to a live virus, a DNA vaccine is more likely to be safe from ERD than vaccine in which the antigen is not presented intracellularly. Though DNA vaccines have been shown to provide protective immunity against a variety of pathogens in various animal models [20,21], their weak immunogenicity in non-human primates and humans [20] suggest a need for an adjuvant to enhance their immuogenicity in humans. The addition of CpG motif or synthetic CpG oligonucleotides (CpG ODN) is a promising adjuvant for DNA vaccines. They have been shown to enhance activation of B cells, natural killer (NK) cells, and monocytes/macrophages to proliferate, mature, and secrete a variety of cytokines, chemokines, and/or immunoglobulins. This activation has been associated with enhanced immunogenicity of DNA vaccines [22,23,24,25,26,27,28]. Four major types of immunostimulatory CpG ODN (A, B, C, and P classes) have been identified based on the differences in structure and the nature of the immune responses they induce in humans [25,29,30,31,32,33]. Previous studies have shown that the C-Class of CpG ODN (CpG-C) is a potent Th1 adjuvant. It combines the biological activities of A- and B-Classes of CpG ODN (CpG-A, CpG-B), and has been studied for use in infectious, allergic, and cancer-related disease [31,34]. It has been also reported that CpG ODN can be directly inserted into the plasmid backbone of DNA vaccine and provide a “built-in adjuvant” which can induce potent antigen-specific immune responses against bacteria and viruses [35,36,37,38,39].

In the current study, we evaluated the ability of built-in CpG-C motif to enhance the immunogenicity of an RSV F protein DNA vaccine. We looked at five or 20 copies of the CpG-C motif cloned into the plasmid encoding RSV F, designated pVAX1-F-CpG5 or pVAX1-F-CpG20, for their ability to enhance the antibody response and direct a Th1 predominant immune response. Our results indicate that addition of the CpG-C motif to the DNA vaccine resulted in a higher titer of serum immunoglobulin (Ig) G and neutralizing antibodies in BALB/c mice, and a more Th1 polarized response. Twenty copies of CpG-C were most effective at decreasing pulmonary pathology and virus replication and weight loss in RSV challenged mice immunized with the DNA vaccine. Thus, we describe a strategy to improve RSV DNA vaccines by inserting the CpG-C motif into the plasmid DNA. With this strategy, a greater Th1 polarized and antigen-specific humoral and cellular immune responses is induced which should facilitate protection from RSV disease.

## 2. Materials and Methods

### 2.1. Preparation of Human CpG-Modified Plasmids

**Figure 1:**
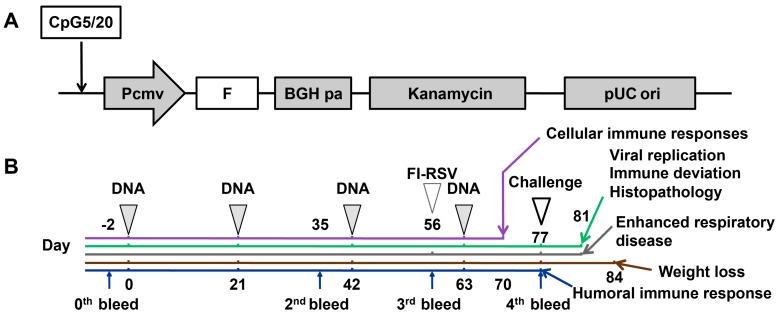
Construction of the modified DNA plasmids with multiple copies of CpG motif, and animal experiment schedule. (**A**) pVAX1 was modified by insertion of five or 20 copies of C-type of CpG ODN in 5′ end of cytomegalovirus (CMV) promoter (Pcmv), and then the codon optimized fusion glycoprotein (F) of human respiratory syncytial virus (RSV) was constructed into this vector, the resulting recombinant plasmids were designated pVAX1-CpG5-F and pVAX1-CpG20-F, respectively; (**B**) groups of five mice were immunized via the intramuscular (i.m.) route in the thigh (quadriceps) with 30 μg of either pVAX1-F, pVAX1-CpG5-F, or pVAX1-CpG20-F, and boosted three times at 3-week intervals. The phosphate buffered solution (PBS, 100 μL/mouse) immunized mice were used as negative control mice (NC). T-cell responses were determined by enzyme-linked immunospot assay (ELISPOT) 7 days after the last immunization (purple line, day 70) and serum binding and neutralizing antibodies were measure at various times points (blue lines, days 2, 35, 56, and 77). For one challenge studies, vaccinated mice were administered 1 × 10^6^ plaque forming units (PFU), or 2 × 10^6^ PFU, by the intranasal (i.n.) route14 days after the last immunization (open arrow, day 77). Lung RSV titer, lung cytokines, and lung histopathology were analyzed 4 days after challenge (green arrow, day 81). Weight loss was assessed for 7 days after challenge. The enhanced respiratory disease (ERD) after formalin-inactivated, alum adjuvanted RSV (FI-RSV) vaccination was assessed in mice challenged with 1 × 10^6^ PFU of RSV 21 days after one dose of FI-RSV vaccine. Histopathology studies for ERD were done at 4 days after challenge.

The C type sense ODN sequence from C274 was 5′-TCGTCGAACGTTCGAGATGAT-3′ (21 bp) [31]. To study the built-in adjuvant activity of human CpG motif in the backbone of the DNA plasmid pVAX1, five or 20 copies of the CpG-C motif were inserted in the plasmid to get CpG-modified plasmids of pVAX1-CpG5 and pVAX1-CpG20. The pVAX1-CpG5 and pVAX1-CpG20 contained an additional 105 bp of dsDNA or 420 bp of dsDNA, respectively. Finally, the codon-optimized gene of RSV F (EF566942) was then introduced into these plasmids and designated pVAX1-CpG5-F or pVAX1-CpG20-F which were used to vaccinate mice as indicated in Figure 1A. The F gene encoded recombinant plasmid, pVAX1-F was used as control for the effect of the addition of the CpG motif. All plasmids were prepared and purified using Endofree Maxi kits (Qiagen, Hilden, Germany) for immunization.

### 2.2. Virus Preparation

Subgroup A RSV Long strain (ATCC, Rockefeller, MD, USA) was propagated in HEp-2 cells (ATCC) in Dulbecco’s Modified Eagle’s Medium (DMEM; Invitrogen, Life Technologies, Carlsbad, CA, USA) supplemented with 2% fetal calf serum (FBS) (HyClone, South Logan, UT, USA), l-glutamine (2 mmol/L), penicillin G (40 U/mL), streptomycin (100 μg/mL) and 0.2% sodium bicarbonate. After the syncytia formation, cells were scraped off and centrifuged for 10 min at 1500× *g*. Supernatants were filtered through a 0.45-mm sterile filter (Merck Millipore, Carolina, NC, USA), and ultracentrifuged at 17,000 rpm through a 10% sucrose (Sigma Aldrich, St. Louis, MO, USA) cushion for 2 h at 4 °C. The pellet was resuspended in 10% sucrose containing phosphate buffered solution (PBS) and stored at 80 °C. The infectivity of the resulting RSV was determined using the immunoplaque assay with slight modifications [40,41]. Briefly, RSV samples were serially diluted (10-fold) in OptiMEM medium (with l-glutamine containing 2% FBS, 2.5% HEPES (1 mol/L) and 1% penicillin G/streptomycin). The RSV dilutions (100 μL) were absorbed onto HEp-2 cells (85% confluency) in 96-well plate in triplicate for 60 min at room temperature (RT). Then, the medium was removed and the cells were washed with DMEM without serum. Finally, 200 μL of DMEM containing 0.9% methyl cellulose (Sigma Aldrich) was added to each well. After 3-day incubation, media was removed and the monolayers were fixed in 95% cold ethanol. Polyvalent mouse anti-RSV antibody (NCLRSV3, Leica Biosystems, Wetzlar, UK) was added (1:1000 dilution) and followed by horseradish peroxidase goat anti-mouse IgG (1:2000 dilution) (Santa Cruz Biotechnology, Dallas, CA, USA). Plaques were visualized by adding 100 μL of tetramethylbenzidine (TMB) substrate solution (Promega, Madison, WI, USA). RSV titers were expressed as plaque-forming units per mL (PFU/mL).

The method to prepare FI-RSV was described previously [10,41]. Briefly, RSV-containing lysates were clarified by centrifugation for 15 min at 550× *g*. The RSV in supernatant was used titrated by immunoplaque assay as described above and was inactivated with formalin (1:4000 dilution) (Sigma-Aldrich) at 37 °C for 72 h with vortexing every 24 h, and then pelleted by ultracentrifugation for 1 h at 17,000 rpm. The resulting pellet was resuspended in 1/25 of the original volume in serum-free DMEM and assayed for protein concentration by bicinchoninic acid (BCA) protein assay kit (Thermo Fisher Scientific, Waltham, MA, USA). Inactivated RSV was adsorbed to alum adjuvant (4 mg/mL) (Thermo Fisher Scientific) and used for immunization.

### 2.3. Vaccination and Challenge of Mice

**Figure 2:**
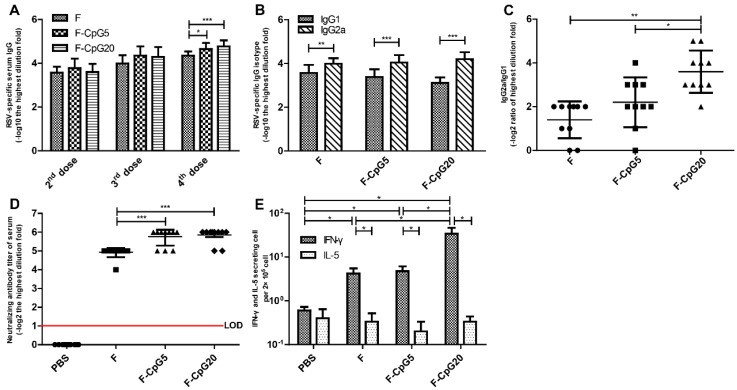
The induced RSV-specific immune responses in mice immunized with CpG-C modified DNA plasmids. BALB/c mice (*n* = 5) were immunized 4 times via i.m. route with either pVAX1-F (30 μg/dose/mouse), pVAX1-F-CpG5 (30 μg/dose/mouse), pVAX1-F-CpG20 (30 μg/dose/mouse), or PBS (negative control, NC, 100 μL/mouse). The serologic results for the two independent experiments were similar so data from both are included in Panels (**A**–**D**). (**A**) Serum total immunoglobulin (Ig) G antibody titers; (**B**) IgG isotype antibody titers; (**C**) the ratio of IgG2a/IgG1 isotype antibody titers depicted in panel (**B**); (**D**) serum neutralizing antibody titers expressed as the reciprocal of the highest serum dilution giving 50% reduction of plaque numbers relative to no antibody control well. The red line is the limit of detection (LOD); (**E**) RSF F-specific ELISPOT immune responses as number of positive spots/2 × 10^5^ splenocytes for interferon gamma (IFN-γ) or inteleukin (IL)-5 at 7 days after the last, 4th, vaccine dose. The data are the mean ± standard deviation (SD). Significance was determined by a Student’s *t*-test in GraphPad Prism, and with each point of data representing one individual animal. * *p* < 0.05, ** *p* < 0.01, *** *p* < 0.001. F: pVAX1-F; F-CpG5: pVAX1-F-CpG5; F-CpG20: pVAX1-F-CpG20.

Specific-pathogen-free female BALB/c mice (Charles River Laboratories, Beijing, China) at 6–8 weeks of age were randomly distributed into groups of five mice. Groups of mice were immunized via the intramuscular (i.m.) route in thigh (quadriceps) with (1) 30 μg of the different DNA plasmids in 0.1 mL of PBS on day 0 and boosted three times at 3 week-interval on days 21, 42, and 63 (Figure 1B); (2) an equal volume of PBS (negative control group); or (3) FI-RSV (equivalent of 1 × 10^6^ PFU RSV/mouse) in 0.1 mL of PBS containing 10% sucrose on day 56. Mice were challenged intranasally with either 1 × 10^6^ PFU or 2 × 10^6^ PFU of RSV in 30 μL at 14 days after the last DNA vaccination or 21 days after FI-RSV vaccination, as indicated in Figure 1B. The body weight changes were monitored for 7 days after 1 × 10^6^ PFU RSV challenge.

### 2.4. Antibody Titer Measurement and Virus Neutralization Assay

Sera from mice in different treatment groups were screened for anti-RSV binding antibody by enzyme-linked immunosorbent assay (ELISA). Briefly, ELISA plates (Corning, New York, NY, USA) were coated with the purified RSV overnight at 4 °C in carbonate coating buffer (pH 9.5). Four fold serial dilution of serum samples were added to the antigen-coated plates in triplicate for 1 h at 37 °C, and the total IgG and IgG1 and IgG2a isotype antibodies were detected with the corresponding horseradish peroxidase (HRP)-conjugated goat anti-mouse IgG, IgG1 or IgG2 antibody (Santa Cruz Biotechnology) at 1:2000 dilution. Serum antibody titers from individual mice were expressed as the reciprocal of the maximum dilution of serum giving an absorbance reading greater than 0.2 absorbance units and 2-fold above the absorbance for serum from the mice of PBS immunization group. To analyze RSV-specific neutralizing antibody titer, serum samples were heat-inactivated at 56 °C for 30 min. Two-fold serial dilutions of sera were prepared in tissue culture medium (MEM with l-glutamine containing 2% FBS, 2.5% HEPES (1 mol/L) and 1% antibiotic/antimycotic) and 50 PFU of RSV virus suspension were incubated with serial dilution of the serum samples at 37 °C for 1 h. Then, 100 µL of the suspension was absorbed onto HEp-2 cells in triplicate. Following incubation for 45 min at RT, the medium was removed and DMEM containing 0.9% methyl cellulose (Sigma Aldrich) was added. Three days later, RSV-specific neutralizing antibody titers were determined as described above. Neutralization titers are expressed as the reciprocal of the serum dilution giving a 50% reduction in PFU relative to the number of plaques without addition of antibody.

### 2.5. Enzyme-Linked Immunospot (ELISPOT) Assays

ELISPOT assays were performed as previously described [42]. Briefly, ELISPOT plates (BD Biosciences, San Jose, CA, USA) were coated overnight at 4 °C with murine interferon gamma (IFN)-γ or interleukin (IL)-5 specific monoclonal antibodies. Splenocytes (2 × 10^5^ cells), collected after 7 days of the last immunization, were added each of triplicate wells and stimulated with 10 μg/mL RSV-F protein. After incubation at 37 °C for 24 h, the cells were then lysed with deionized (DI) water, and the plates were incubated at RT with biotinylated IFN-γ or IL-5 antibody for 2 h and peroxidase-labeled streptavidin for another 1 h. After washing with PBS, 100 μL of the final substrate solution was added to each well, and spot development was monitored. The plates were washed with DI water to stop the reaction. IFN-γ and IL-5 spot-forming cells (SFC) were counted automatically using an ELISPOT reader (BD Biosciences) and analyzed using ImmunoSpot image analyzer software v4.0 (BD Biosciences).

### 2.6. Quantification of RSV Titers and Cytokines in Lungs with Real-Time Quantitative Polymerase Chain Reaction (RT-qPCR) and RT-PCR, Respectively

The mice were sacrificed on day 4 after challenge. The right lung was weighed, placed in sterile MEM (1 mL/0.1 g lung), and homogenized with a glass tissue grinder. The homogenates were centrifuged (10,000× *g* for 1 min) and 100 μL of lung homogenate supernatant was used to isolate viral RNA by Trizol reagent (Invitrogen) according to the manufacturer’s instructions. Using 30 μL of elution buffer to elute the viral RNA and reverse transcribing the RNA to complementary DNA (cDNA) with Reverse Transcription System (Promega). The cDNA was quantified using the SuperReal PreMix procedure (Tiangen Biotech, Beijing, China) with the sense primer combined with RSV-N gene, RSA-1 5′-AGATCAACTTCTGTCATCCAGCAA-3′; antisense primer, RSA-2 5′-GCACATCATAATTAGGAGTATCAAT-3′ [41,43,44]. cDNA standard was prepared by cloning the RSV-N gene into the pMD18-T vector (TaKaRa, Tokyo, Japan). The samples were quantified by the standard under the following conditions: 15 min at 95 °C, and 40 cycles of 15 s at 95 °C and 1 min at 60 °C. For the cytokine analysis, the primers for Th1 (IFN-γ and tumor necrosis factor alpha (TNF-α)), Th2 (IL-4 and IL-5) and Th17 (IL-17) cytokines detection were synthesized and the quantification of the cytokine messenger RNA (mRNA) was carried out using RT-PCR as the previous study [45]. The data were normalized relative to glyceraldehyde phosphate dehydrogenase (GAPDH) by using the following formula: relative mRNA expression = 2^−ΔΔ*C*t^, where Ct is the threshold cycle value [46,47,48] and PBS immunization and followed by RSV-challenged mice were used as the control.

### 2.7. Lung Histopathology of the BALB/c Mice Challenged with RSV Following Immunization

Left lungs from immunized mice were harvested on day 4 after RSV challenge and fixed in 10% neutral buffered formalin, embedded in paraffin in the dorsoventral position. Subsequently, 5 μm-thick sections were obtained and stained with hematoxylin and eosin (H & E). Using a semiquantitative scale (0 to 4) (0 = absent and 4 = maximum/severe), a board-certified pathologist evaluated all slides for inflammatory infiltration around peribronchial and/or peribronchiolar, perivascular, and interstitial regions. FI-RSV immunized BALB/c mice were used as control for vaccine-enhanced pulmonary pathology.

### 2.8. Statistical Analyses

Statistical analyses were performed with GraphPad Prism 5 (GraphPad Software, La Jolla, CA, USA). Correlations were calculated by using an unpaired, two tailed Student’s *t*-test. For all tests only data resulting in *p*-values < 0.05 were regarded as statistically significant.

### 2.9. Ethical Approval

This study was conducted with the approval by the Institutional Animal Care and Use Committees at Tsinghua University (No. 14-DZJ2, approved on 8 October 2014).

## 3. Results

### 3.1. RSV-Specific Immune Responses

To investigate the in vivo activity of CpG-modified plasmids encoding codon optimized RSV F, the induced RSV-specific humoral immune responses by these CpG-modified DNA vaccines were analyzed and compared with the control plamid of pVAX1-F (Figure 2A–D). After the 4 doses, significantly higher titer of RSV-specific serum antibody was induced with the pVAX1-CpG5-F or the pVAX1-CpG20-F than with the pVAX1-F vaccine (*p* < 0.01). All of the immunized mice displayed the ratios of IgG2a/IgG1 > 1 indicating induction of a Th1-type humoral immune response (Figure 2B). Of note the pVAX1-CpG20-F vaccine induced a significantly higher IgG2a/IgG1 isotype ratio compared with pVAX1-F (*p* < 0.01) and pVAX1-CpG5-F (*p* < 0.05) (Figure 2C).

Potent neutralizing antibodies are essential to control RSV replication in the lower respiratory tract. Our results demonstrated that pVAX1-CpG5-F and pVAX1-CpG20-F vaccines induced similar levels of neutralizing antibodies that were significantly higher than pVAX1-F vaccine (*p* < 0.001) (Figure 2D).

Based on the serologic studies, addition of the CpG motif not only enhanced serum and neutralizing antibodies, but also induced greater Th1 polarized responses in mice. The most Th1 polarized antibody response, i.e., highest IgG2A/IgG1 ratio, was seen with 20 copies of CpG motif.

The T-cell immune responses are an important factor in virus clearance. Therefore, we investigated the number of IFN-γ and IL-5 secreting cells in splenocytes stimulated with F protein by ELSPOT before challenge. The pVAX1-CpG20-F group produced higher numbers of IFN-γ secreting cells than either pVAX1-CpG5-F, pVAX1-F, or PBS groups (*p* < 0.05). The number of IL-5 secreting cells was low for all groups (Figure 2E). These results showed that RSV F-specific T-cell responses were induced by all the F protein DNA vaccines, with the pVAX1-CpG20-F vaccine inducing the most robust IFN-γ, or Th1-biased, response. None of the vaccines induced a significant IL-5 indicating a lack of a Th2-biased T-cell response.

### 3.2. Protection against RSV Infection

**Figure 3:**
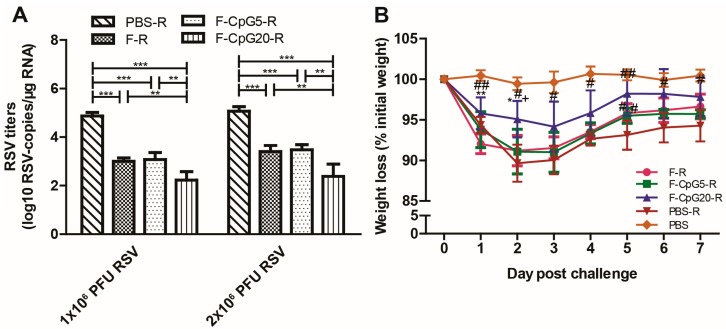
Lung virus titers and body weight changes in vaccinated mice after RSV challenge. BALB/c mice (*n* = 5) were immunized 4 times via (i.m. route with either pVAX1-F (30 μg/dose/mouse), pVAX1-CpG5-F (30 μg/dose/mouse), pVAX1-CpG20-F (30 μg/dose/mouse), or PBS (NC, 100 μL/mouse). (**A**) The vaccinated mice were intranasally challenged with 1 × 10^6^ (experiment 1) or 2 × 10^6^ (experiment 2) PFU at 14 days after the 4th vaccination. RSV copies per microgram RNA from lung homogenates detected by real-time quantitative polymerase chain reaction (RT-qPCR) and expressed as log10 RSV-copies/μg RNA; (**B**) the body weight changes were monitored and calculated daily as a percentage of starting weight for 7 days post 1 × 10^6^ PFU RSV challenge, the symbol of either #, +, or * indicated significant difference (*p* < 0.05) compared with mice in the group of PBS-R, F-R, or F-CpG5-R; ## indicated *p* < 0.01. Data showed mean ± SD, analyzed by two-way analysis of variance (ANOVA) in GraphPad Prism, and each point of data represented an individual animal. ** *p* < 0.01, *** *p* < 0.001. F: pVAX1-F; F-CpG5: pVAX1-F-CpG5; F-CpG20: pVAX1-F-CpG20; R: challenge.

To illustrate the capability of the mice immunized by CpG-modified DNA vaccine candidates to provide protection against RSV infection, RSV load in lungs of immunization mice was measured at 4 days after 1 × 10^6^ PFU, or 2 × 10^6^ PFU of RSV challenge via i.n. route by RT-qPCR. As shown in Figure 3A, compared to the negative control group (PBS-R), all three of the plasmid vaccines provided effective protection from RSV infection (*p* < 0.001), with mice vaccinated pVAX1-CpG20-F having the lowest level of RSV RNA after either RSV challenge dose.

Body weight change is another important parameter in assessing protective efficacy after RSV challenge in a mouse model. Substantial weight loss was observed in PBS immunized mice after RSV challenge (>10%) compared to the pVAX1-CpG20-F (<6%) and the pVAX1-CpG5-F and pVAX1-F-immunized mice (ca. 9%) (Figure 3B). Though all vaccines groups were associated with less weight loss, the pVAX1-CpG20-F group showed the least and shortest duration of weight loss. Collectively, these results suggested pVAX1-CpG20-F was the most effective of the DNA vaccine for protecting from post challenge RSV disease.

### 3.3. Pulmonary Pathology after RSV Challenge

**Figure 4:**
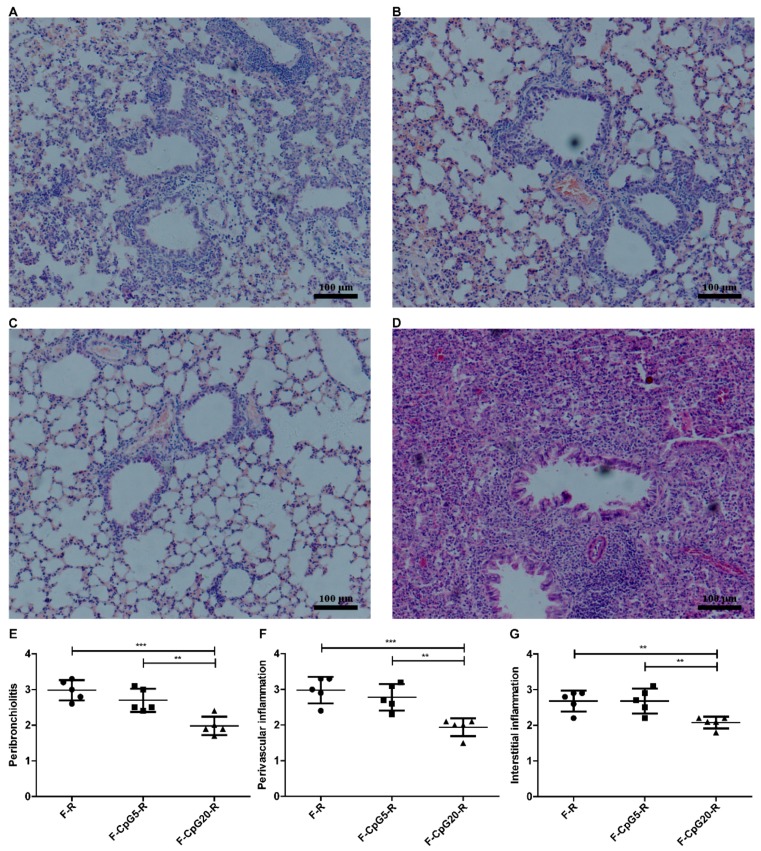
Histopathology analysis of Hematoxylin Eosin (H & E) stained lungs from immunized mice after RSV challenge. BALB/c mice (*n* = 5) were immunized 4 times via i.m. route with either pVAX1-F (30 μg/dose/mouse), pVAX1-CpG5-F (30 μg/dose/mouse), or pVAX1-CpG20-F (30 μg/dose/mouse), mouse). In addition, the mice, immunized by single injection of FI-RSV (equivalent of 1 × 10^6^ PFU RSV/mouse), was used for the analysis of ERD. The vaccinated mice were intranasally challenged with RSV of 1 × 10^6^ PFU at 14 days after the last DNA immunization or 21 days after FI-RSV immunization. The lung tissues (**A**–**D**) from F (**A**); F-CpG5 (**B**); F-CpG20 (**C**); or FI-RSV (**D**) immunization mice were collected at day 4 post RSV challenge and prepared for light micrographic analyses of pulmonary histopathology by H & E stain. The H & E stained lungs were scored (**E**–**G**) for inflammation with semi-quantitative scale from 0 to 4 (0 = absent and 4 = maximum/severe); including the inflammation scores of the peribronchiolar region (**E**); the perivascular region (**F**); and the interstitial region (**G**). Data showed mean ± SD, analyzed by one-way ANOVA with non-parametric test, and each point of the data represented an individual animal. ** *p* < 0.01, *** *p* < 0.001. F: pVAX1-F; F-CpG5: pVAX1-F-CpG5; F-CpG20: pVAX1-F-CpG20.

It is expected that an RSV vaccine with Th1-biased and potent neutralizing antibody response will have less pulmonary pathological changes after viral challenge and not show the ERD type of changes seen in FI-RSV immunized mice. As shown in Figure 4A–D, we found reduced lung histopathology in immunized mice after RSV challenge with all of the DNA vaccines compared to FI-RSV vaccine. The pVAX1-CpG20-F-immunized and RSV-challenged mice had a lower degree of peribronchial, perivascular, and interstitial inflammation than RSV challenged mice immunized with pVAX1-CpG5-F (*p* < 0.01) or pVAX1-F (*p* < 0.001 or *p* < 0.01) (Figure 4E–G). There was not a significant difference in the lung pathology between mice immunized with pVAX1-CpG5-F and those immunized with pVAX1-F. These results indicated that pVAX1-CpG20-F was better in preventing lung histopathology than pVAX1-CpG5-F or pVAX1-F and this effect was dependent on copy number of CpG motif in plasmid.

### 3.4. Pulmonary Cytokine mRNA Levels after Challenge

**Figure 5:**
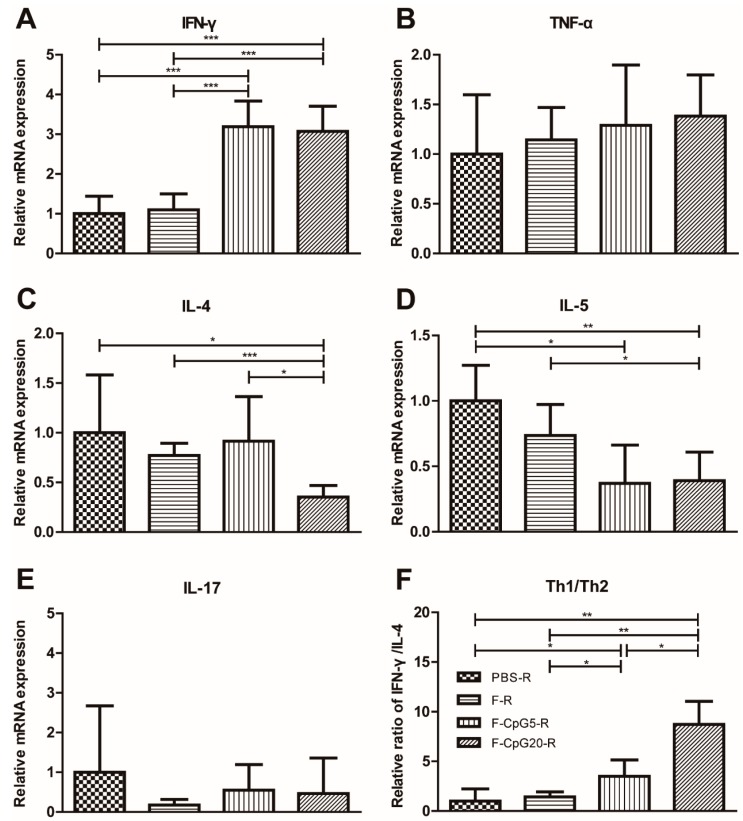
Cytokine production in lungs of immunized mice after 4 day of RSV challenge. BALB/c mice (*n* = 5) were immunized 4 times via i.m. route with either pVAX1-F (30 μg/dose/mouse), pVAX1-CpG5-F (30 μg/dose/mouse), pVAX1-CpG20-F (30 μg/dose/mouse), or PBS (NC, 100 μL/mouse). The vaccinated mice were intranasally challenged with 1 × 10^6^ PFU of RSV after 14 days of last immunization. The messenger RNA (mRNA) levels of IFN-γ (**A**); tumor necrosis factor alpha (TNF-α) (**B**); IL-4 (**C**); IL-5 (**D**); and IL-17 (**E**) were tested with RT-PCR and were normalized relative to glyceraldehyde phosphate dehydrogenase (GAPDH). The formula (mRNA expression = 2^−ΔΔ*C*t^, *C*t is the cycle threshold) was used to calculate the cytokine secretion level of each group; (**F**) Ratios of IFN-γ/IL-4 were calculated to illustrate the polarization of immunized mice upon challenge. Data showed mean ± SD, analyzed by two-way ANOVA in GraphPad Prism. * *p* < 0.05, ** *p* < 0.01, *** *p* < 0.001. F: pVAX1-F; F-CpG5: pVAX1-F-CpG5; F-CpG20: pVAX1-F-CpG20; R: challenge.

The mice, vaccinated with FI-RSV, were induced ERD, which resulted in an increase in cytokine expression and the development of Th2 polarized immune response. In order to further evaluate the safety of these vaccine candidates, the mRNA for Th1-, Th2- and Th17-type cytokine was measured in lung homogenates by RT-PCR at 4 days after RSV challenge in the immunized mice. IFN-γ mRNA was detected at the higher levels in pVAX1-CpG5-F or pVAX1-CpG20-F vaccinated mice compared to mice vaccinated by pVAX1-F or in negative control group (Figure 5A). Another Th1-associated cytokine, TNF-α, did not show significant differences among these groups (Figure 5B). IL-4, associated with a Th2-type response, was remarkably decreased in pVAX1-CpG20-F group compared to the other groups (Figure 5C), while IL-5 mRNA was significantly lower for both pVAX1-CpG5-F and pVAX1-CpG20-F vaccinated mice (Figure 5D). The ratio of Th1 to Th2 cytokines, i.e., IFN-γ/IL-4 mRNA, was considerably higher after pVAX1-CpG5-F and pVAX1-CpG20-F vaccinations (Figure 5F). None of the vaccinated mice showed a significant IL-17 response (Figure 5E). These findings showed that inclusion of multiple copies of the CpG motif in a plasmid DNA vaccine both enhanced the immune response and directed a more favorable Th1-type response in mice.

## 4. Discussion

Despite more than 50 years of research, there is no licensed vaccine available against RSV with both safety and efficacy being a challenge. In this study, we show that addition of multiple CpG motif into a DNA RSV F plasmid vaccine helps to address both issues. The ERD associated with the FI-RSV vaccine given to young children and some other animal and human studies suggests that a Th1 versus a Th2 biased response is favored from the safety perspective. Our data show that addition of multiple CpG motif, especially the higher number 20, resulted in an enhanced shift toward a Th1-biased antibody response and T-cell memory response. In our study, multiple copies of CpG-modified DNA vaccines increased IgG2a/IgG1 isotype ratio and produced higher numbers of IFN-γ secreting splenocytes 7 days after the last vaccination and 7 days before challenge. Furthermore, the mRNA profile in lungs at 4 days post challenge also showed a Th1-biased inflammatory response as indicated by the high level of IFN-γ mRNA relative to IL-4 mRNA levels in mice receiving the CpG-modified DNA vaccines. The lower level of IFN-γ mRNA and higher levels of IL-4 and/or IL-5 for mice vaccinated with the F alone DNA vaccine, pVAX1-F, or mock vaccine support the shift to a Th1-type response. Additionally, the mice receiving the CpG-modified vaccines, especially the 20 copies vaccine, had less pulmonary pathology, virus replication, and weight loss.

DNA vaccines are attractive for RSV vaccines since they express RSV proteins intracellularly without the risks associated with a replicating virus. Intracellular expression of antigens is comparable to that for a live virus, which is not associated with ERD and likely to be safe from ERD risk. DNA vaccines similar to the pVAX1-F in this study have previously been shown to elicit Th1 polarized immune responses and reduce pulmonary inflammation in mice [49,50,51]. CpG ODN has previously been studied with RSV vaccines and found, in animal studies, to be a safe and effective adjuvant [52,53,54]. Our study shows that the addition of CpG ODN directly to the backbone of DNA vaccine is an effective adjuvant that directs toward immune responses likely to be safe and effective, i.e., it induced higher levels of binding and neutralizing antibodies, more F protein-specific responding T cells, and a more Th1 biased response. We also noted that increasing the number CpG motif increased this adjuvant effect. Thus, building multiple copies of CpGs into an RSV DNA vaccine should improve both its safety and efficacy.
